# AIGen: an artificial intelligence software for complex genetic data analysis

**DOI:** 10.1093/bib/bbae566

**Published:** 2024-11-16

**Authors:** Tingting Hou, Xiaoxi Shen, Shan Zhang, Muxuan Liang, Li Chen, Qing Lu

**Affiliations:** Department of Experimental Statistics, Louisiana State University, 45 Martin D. Woodin Hall, Baton Rouge, LA 70802, United States; Department of Mathematics, Texas State University, 601 University Drive San Marcos, TX 78666, United States; Department of Biostatistics, University of Florida, 2004 Mowry Road, Gainesville, FL 32611, United States; Department of Biostatistics, University of Florida, 2004 Mowry Road, Gainesville, FL 32611, United States; Department of Biostatistics, University of Florida, 2004 Mowry Road, Gainesville, FL 32611, United States; Department of Biostatistics, University of Florida, 2004 Mowry Road, Gainesville, FL 32611, United States

**Keywords:** neural networks, human genome, complex relationships, MINQUE, batch training

## Abstract

The recent development of artificial intelligence (AI) technology, especially the advance of deep neural network (DNN) technology, has revolutionized many fields. While DNN plays a central role in modern AI technology, it has rarely been used in genetic data analysis due to analytical and computational challenges brought by high-dimensional genetic data and an increasing number of samples. To facilitate the use of AI in genetic data analysis, we developed a C++ package, AIGen, based on two newly developed neural networks (i.e. kernel neural networks and functional neural networks) that are capable of modeling complex genotype-phenotype relationships (e.g. interactions) while providing robust performance against high-dimensional genetic data. Moreover, computationally efficient algorithms (e.g. a minimum norm quadratic unbiased estimation approach and batch training) are implemented in the package to accelerate the computation, making them computationally efficient for analyzing large-scale datasets with thousands or even millions of samples. By applying AIGen to the UK Biobank dataset, we demonstrate that it can efficiently analyze large-scale genetic data, attain improved accuracy, and maintain robust performance. **Availability**: AIGen is developed in C++ and its source code, along with reference libraries, is publicly accessible on GitHub at https://github.com/TingtHou/AIGen.

## Introduction

With rapidly evolving high-throughput technologies, large-scale genetic studies have been conducted to accelerate the gene discovery process [1, 2]. While the vast amounts of genetic data collected from these studies hold great promise for uncovering novel variants predisposing to human complex diseases, the massive and high-dimensional data bring analytical and computational challenges to data analysis. Moreover, most human diseases are likely influenced by multiple variants in a complicated manner. This complexity, however, has been paid little attention to by existing methods, adding another layer of difficulty to the discovery process. There is a pressing need to develop advanced methods and software to address the challenges encountered in large-scale genetic data analysis.

Advanced statistical methods have been developed to analyze high-dimensional genetic data, including methods developed based on linear mixed methods (LMM) and functional linear models (FLM). LMM-based methods, such as the sequence kernel association test [3] and the genome-wide complex trait analysis (GCTA) [4], have been widely used in high-dimensional genetic data analysis. Instead of considering genetic effects as fixed effects, LMM-based methods model the overall genetic effects as random effects, which could substantially reduce the number of parameters and make them applicable to thousands or even millions of variants. FLM-based methods [5, 6], which model genetic effects as functions, have several advantages for genetic data analysis, such as using information from nearby loci, reducing the number of parameters, and addressing the missing data issue. While these methods have many advantages and have been widely used in high-dimensional genetic data analysis, most of them are designed for modeling linear and additive genotype-phenotype relationships, which limits their capability to model complex relationships, such as interactions.

These methods can be enhanced by utilizing state-of-the-art artificial intelligence (AI) technology. Deep neural networks (DNN) [7] have been the driving force in modern AI technology and have been widely used in areas such as computer vision and genomics [8, 9]. Given the amazing performance of DNN in these areas and its capability of dealing with interactions [10], it is expected that it could play an important role in genetic data analysis. In our prior work, we integrated DNN into LMM and FLM, and developed two innovative neural network frameworks: kernel neural networks and functional neural networks [11–13]. Both methods exhibit remarkable performance in capturing non-linear and non-additive genetic effects (e.g. interactions), while having the same advantages as LMM and FLM (e.g. being applicable to millions of variants or using information from nearby loci). We develop these two new frameworks into a powerful and computationally efficient tool that researchers can apply in their studies. In this paper, we first provide a brief overview of the two frameworks and then apply the software to genotypes and phenotypes (i.e. pack-years of smoking and diabetes), from the UK Biobank (UKB) database.

## Methods

With the advance of technology and reduced costs, we are able to produce millions of single-nucleotide polymorphisms (SNPs) and comprehensively evaluate their roles in complex human diseases. While the vast amounts of genetic data collected from large-scale studies hold great promise for new discovery, the high-dimensional genetic data (i.e. thousands or even millions of variants), and the complex genetic structure, the sophisticated genotype-phenotype relationships (e.g. interactions), and the increased sample size bring tremendous analytic and computational challenges. To address these challenges and facilitate high-dimensional genetic data analysis, we develop AIGen, an AI-based tool with two modules, kernel neural network (KNN) and FNN. Both modules utilize new neural network structures to consider complex relationships and classic statistical frameworks (i.e. LMM and FLM) to handle high-dimensional genetic data.

### Kernel neural network

KNN is designed for complex genetic data analysis involving thousands or millions of SNPs. It utilizes kernel matrices to handle a large number of SNPs, and model the overall effect of all available markers as a random effect via a LMM framework, which substantially reduces the number of parameters and complexity of the neural network structure. Based on the input kernel matrices, KNN further builds a kernel neural network to model the complex non-linear and non-additive genetic effects (e.g. interactions). Minimum norm quadratic unbiased estimation (MINQUE) and batch training are implemented in KNN to accelerate the computation, making it applicable to datasets with a large number of samples.

In KNN, the phenotype $\boldsymbol{y}$ is modeled via a random effect model with genetic data $\boldsymbol{X}$,


\begin{align*} \boldsymbol{y|Z,a} &\sim \mathcal{N}_{n} \left(\boldsymbol{Z}\boldsymbol{\beta}+\boldsymbol{a},\phi \boldsymbol{I}_{n} \right) \nonumber \\ \boldsymbol{a}|\boldsymbol{u_{1}}, \dots, \boldsymbol{u_{m}} &\sim \mathcal{N}_{n}\left(\boldsymbol{0},\sum_{j=1}^{J} \tau_{j} \boldsymbol{H}_{j}(\boldsymbol{U})\right) \nonumber \\ \boldsymbol{u_{1}}, \dots, \boldsymbol{u_{m}} &\sim \mathcal{N}_{n}\left(\boldsymbol{0},\sum_{l=1}^{L} \xi_{l} \boldsymbol{K}_{l}(\boldsymbol{X})\right) \nonumber\end{align*}

where $\boldsymbol{Z}$ is the design matrix for fixed effects $\boldsymbol{\beta }$ (e.g. age). The covariance matrix of the random effect $\boldsymbol{a}$ is a positive combination of hidden kernel matrices $\boldsymbol{H}_{j}(\boldsymbol{U})$, which are constructed based on hidden units $\boldsymbol{u}_{1},\ldots ,\boldsymbol{u}_{m}$ and non-linear kernels (e.g. polynomial kernels). The covariance matrix of the hidden units $\boldsymbol{u_{i}}$ is a positive combination of $\boldsymbol{K}_{l}(\boldsymbol{X})$s. $\boldsymbol{K}_{l}(\boldsymbol{X}), l=1, \cdots , L$ are input kernel matrices constructed based on genetic markers. For example, we can define $\boldsymbol{K}(\boldsymbol{X})=p^{-1}\boldsymbol{XX}^{T}$, which is known as the product kernel or genetic relationship matrix [14].

**Figure 1:**
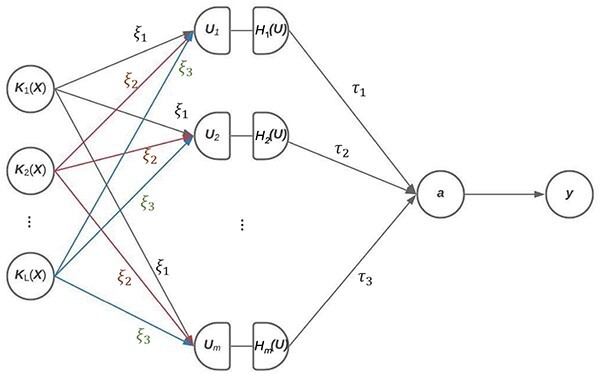
An illustration of the hierarchical structure of the kernel neural network model.

Figure 1 illustrates the hierarchical structure of KNN. As we can see from Fig. 1, the hierarchical structure of KNN is very similar to that of classical DNN, except that inputs and hidden units are now both kernel matrices. By combining the strengths of both LMM and DNN, KDNN has several nice features. First, by modeling the overall effect of all available markers instead of the individual effect of each marker, KNN can handle datasets with millions of markers with substantially reduced parameters. This addresses the limitation of classical DNN, which is computationally prohibitive for such a large number of markers. Meanwhile, the model complexity is also greatly reduced, which alleviates the overfitting issue. Second, KNN can use hidden kernel matrices to capture non-linear and non-additive genetic effects, and therefore is able to model the complex relationships (e.g. interactions).

### Functional neural network

Although KNN is a powerful and computationally efficient tool for complex genetic data analysis, its application is confined primarily to the measurement of a disease phenotype (e.g. diabetes status), which is typically a scalar variable. To analyze various types of phenotypes in the form of vectors or matrices (e.g. the progression of disease measured over time or neuroimaging phenotypes), we also developed a new FNN method. FNN uses a series of basis functions to model genetic data and disease phenotypes, and further builds multi-layer functional neural networks to capture their interactive and complex relationships. FNN has a built-in facility to account for the underlying correlation structures of genetic and phenotype data, and can be used to analyze different types of phenotypes, including high-dimensional phenotypes (e.g. disease outcomes measured over time).

**Figure 2:**
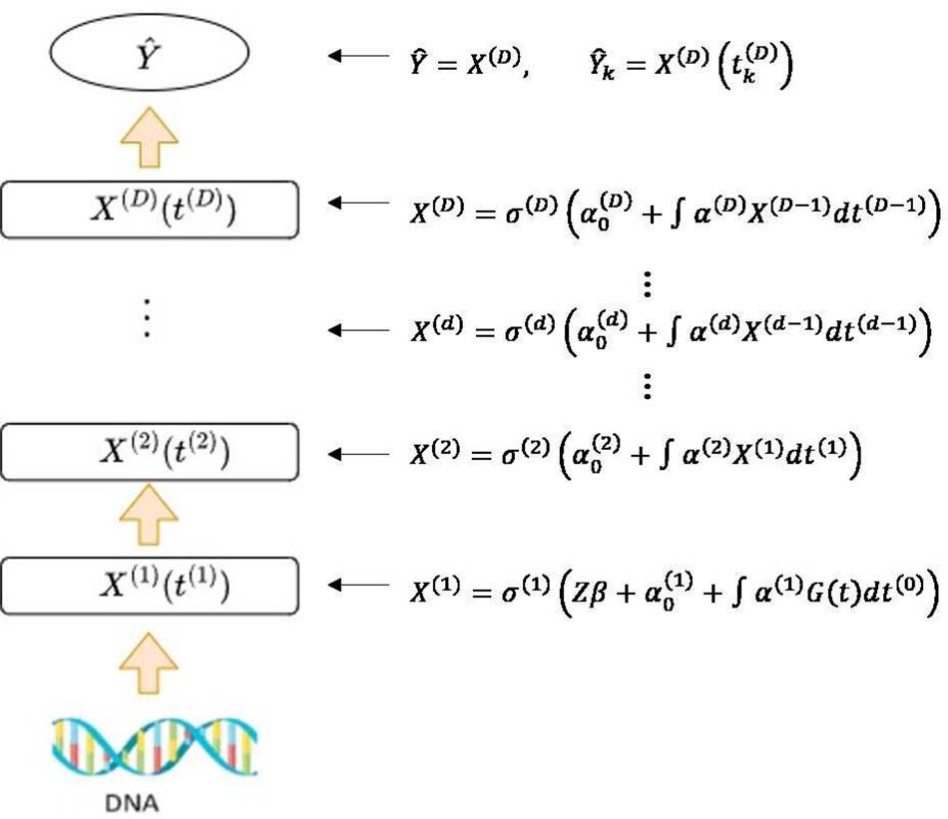
An illustration of the hierarchical structure of the functional neural network model.

Figure 2 shows the hierarchical structure of the FNN model, where $t^{(d)}$ serves as the coordinate system of the $d$th layer; $\sigma $ is an activation function, and $\boldsymbol{Z}$ is the covariate vector and $G(t)$ is the genetic variant function. When $Y$ is a scalar phenotype, $\alpha _{0}^{(D)}$ is a scalar and $\alpha ^{(D)}$ is a univariate function. When $Y$ is a vector, $\alpha _{0}^{(D)}$ is a univariate function and $\alpha ^{(D)}$ is a bivariate function. To estimate the parameters in the model, we use squared error or cross-entropy loss for continuous or categorical phenotypes, respectively. A roughness penalty term is added to the loss function to control the model complexity. Based on FLM and DNN, FNN has several remarkable features: (1) it can be used to analyze different types of phenotypes, including high-dimensional phenotypes; (2) it considers the underlying data structure, such as linkage disequilibrium (LD), for improved performance while reducing the model complexity; (3) it uses the hierarchy structure of function neural networks to capture abstract and complex features from data; and (4) it has the advantage of handling measurement errors and missing data by fitting data with functions and utilizing the information from adjacent markers and phenotype measurements.

### Implementation

AIGen is a versatile C++ software designed to implement two novel types of neural networks, namely KNN and FNN, for modeling complex genotype-phenotype relationships. The software is available for both Windows and Linux operating systems, ensuring compatibility across different platforms. A key feature of AIGen is its implementation of OpenMP parallel processing capabilities, which enable efficient and systematic utilization of multi-core processors for faster computations. Moreover, AIGen has been designed with memory efficiency, allowing it to handle high-dimensional genetic data without consuming excessive system resources. To further boost the training process, the software also offers GPU support, leveraging the power of parallel processing in modern graphics cards to significantly accelerate the model training and evaluation phases. With its comprehensive features and efficient implementation, AIGen is poised to become a valuable AI tool for complex genetic data analysis.

We have integrated the KNN and FNN modules into AIGen, which can be accessed with the ‘–knn’ and ‘–fnn’ commands, respectively. The choice of the module depends on the types of data and research objectives. AIGen facilitates efficient data management by accommodating various data formats commonly used in genetics research. The input data can be either[15] binary format or text format with the commands ‘–bfile’ or ‘–file’, which are widely used in genetics research and are efficient for data storage. Covariate and phenotype files are commonly saved as text formats, with the initial two columns being the family ID and individual ID and subsequent columns being phenotypes or covariates. To facilitate the data process, AIGen’s KNN module has the capacity to create a kernel matrix from the input genetic data by using a range of inbuilt kernel functions, such as the product kernel, Gaussian kernel, and polynomial kernel. The software also facilitates the use of binary files like GCTA (*.grm.bin, *.grm.id) [4] to input kernel matrices, thereby providing users with the option to leverage custom kernel matrices based on their prior knowledge, thus bolstering the versatility and adaptability of AIGen for complex genetic data analysis.

The AIGen software’s output provides a set of measurements aimed at evaluating the performance of the training data for both continuous and categorical phenotypes. In the case of continuous phenotypes, it outputs the Mean Squared Error (MSE) and correlation coefficients, which measure the accuracy performance of the formed models. For binary or multi-class phenotypes, AIGen produces a miss-classification rate and an Area Under the Curve (AUC) value, evaluating the classification and discriminatory accuracy of the model.

## Result and discussion

To demonstrate the use of AIGen, we applied AIGen to a large-scale UKB dataset[16]. UKB is a population-based prospective cohort of $\sim $500,000 individuals recruited in the United Kingdom who were aged 40–69 years. UKB contains a wealth of data on detailed health-related information, genome-wide genotype data and whole-exome sequencing data. For illustration purposes, we selected two scalar phenotypes, pack-years of smoking and diabetes, from UKB. Pack-years of smoking quantifies long-term cigarette usage, which is calculated by dividing the daily number of cigarettes smoked by twenty and then multiplying by the years the individual has smoked. Diabetes is a binary phenotype, denoting the presence or absence of the condition. Besides pack-years of smoking and diabetes, an additional application to systolic blood pressure can be found in supplementary data. Prior to the data analysis, we followed the approach used in our previous publication [13] and performed a stringent quality control procedure. The quality control procedure was performed using PLINK 1.9 [15]. After the quality control process, the final dataset for the analysis comprised 516 429 candidate SNPs and 320 021 individuals, among which 97 318 and 239 938 individuals have pack-years of smoking and diabetes information, respectively.

We first used the KNN module within the AIGen software for whole-genome analysis of the processed UKB dataset by dividing the dataset into a training set (80%) and a testing set (20%). The training set has 77 854 individuals with pack-years of smoking information and 191 950 individuals with diabetes information, while the testing set has 19 464 and 47 988 individuals with pack-years of smoking and diabetes information, respectively. To fit the KNN model, we constructed an input linear kernel on the whole genome-wide data and specified a polynomial kernel as the hidden kernel. Important covariates, including the UKB assessment center, age at recruitment, sex, and top 10 principal components, were also adjusted in the KNN model. We adopted the variance components estimated from MINQUE(0) as initial values and used MINQUE to estimate the variance component parameters. Depending on the phenotype under consideration, the MSE or misclassification, as well as correlation or area under the curve (AUC), were used to assess the accuracy performance of the KNN models. To further illustrate the computation efficiency and accuracy performance of batched KNN methods, we conducted analyses by dividing the data into different numbers ( e.g. 20) of subsets (i.e. batches), running KNN on each batch, and averaging the results to obtain the final model.

Table 1 shows the accuracy performance of KNN with different batch sizes. The MSE of the KNN model with all samples is 0.8261, whereas the MSE estimates of the KNN models with batch numbers of 10, 20, 25, 50, and 100 are 0.8263, 0.8271, 0.8323, 0.8262, and 0.8262, respectively. Similarly, the correlation estimates of the KNN models with batch numbers of 1, 10, 20, 25, 50, and 100 are 0.4225, 0.4224, 0.4215, 0.4153, 0.4224, and 0.4224, respectively. Overall, KNN with batch learning has comparable performance with KNN without batch learning (i.e. Batch=1), while the computation time for KNN with batch training has been substantially reduced.

**Table 1:** The accuracy and computational performance of KNN with different batch numbers when analyzing pack years of smoking and diabetes data from UKB

	**Size**	**Batch**	**Batch sizes**	**Time (s)**	**Mem(GB)**	**MSE/MCE**	**Cor/AUC**
**Pack years of smoking**	97318	1	97318	26 165.60	126.09	0.8261	0.4225
		10	9731	703.72	53.01	0.8263	0.4224
		20	4865	404.94	53.01	0.8271	0.4215
		25	3892	386.71	53.01	0.8323	0.4153
		50	1946	360.82	53.01	0.8262	0.4224
		100	973	344.80	53.01	0.8262	0.4224
**Diabetes**	239 938	1	239 938	351 767.00	764.17	0.0521	0.6887
		10	23 993	8225.65	322.67	0.0521	0.6882
		20	11 996	3598.11	321.88	0.0521	0.6881
		25	9597	3207.65	321.88	0.0521	0.6884
		50	4798	2495.80	321.88	0.0521	0.6886
		100	2399	2301.19	321.88	0.0521	0.6890

The results of diabetes show similar trends with those of pack-years of smoking. Instead of using MSE and correlation, the miss-classification rate and the area under the ROC curve (AUC) are used to evaluate the performance of binary diabetes phenotype. The AUC of the KNN models with the batch numbers of 10, 20, 25, 50, and 100 are 0.6882, 0.6881, 0.6884, 0.6886, and 0.6890, respectively, which is comparable to that of the KNN model with the whole sample (i.e. AUC=0.6887).

The computational efficiency of KNN is also summarized in Table 1. When analyzing the pack-years of smoking data with 97 318 individuals, the software used 126.09 GB for both training and testing procedures. By using batch training, the memory requirement was significantly reduced to 53.01 GB. A similar trend was observed for the analysis of Diabetes data involving 239,938 individuals, where the memory usage dropped from 764.17 GB to approximately 322 GB with the batch training. In addition, the batch method substantially improved the computational efficiency of the analysis. The analysis of pack-years of smoking took over 7 hours (26 165.6 seconds) without batching training, but was dramatically reduced to around 5 minutes (344.798 seconds) when 100 batches were used. The computational advantage became even more pronounced in the analysis of Diabetes data. The analysis without batching training took over 4 days (351 767 seconds), but the analysis with 100 batches was accomplished in just about 38 minutes (2301.19 seconds).

We further demonstrated the use of the FNN module for candidate gene analysis. For this illustration, we applied FNN to two well-known candidate genes,*CHRNA5*[17] and *TCF7L2*[18] genes, associated with smoking and diabetes, respectively. Using Ensembl and PLINK, we extracted 22 SNPs in *CHRNA5* and 59 SNPs in *TCF7L2*, from UKB genetic data, and imputed missing values. The extracted data was then divided into training (80%, with further division into sub-training [80%] and validation [20%] data) and testing (20%). We used fourth-order B-spline basis functions, two hidden layers with 100 and 50 knots, and 10 000 epochs to train FNN. The optimization algorithm employed was ‘adam’, and the optimal penalty parameter $\lambda $ was determined based on validation loss. Depending on the type of phenotypes, the models’ performances were evaluated on the testing data by calculating the MSE/misclassification and correlation/AUC. For comparison purposes, we also applied a traditional neural network with a similar structure, KNN and GCTA to the same data.

We used FNN, NN, KNN, and GCTA to predict pack-years of smoking and diabetes risks. MSE and correlation calculated from both methods for pack-years of smoking are summarized in Table 2. The results from testing data show that FNN outperforms the other three methods with a lower MSE and a higher correlation. MSE and correlation values from FNN in both training and testing datasets are more similar than those from NN, suggesting a more robust performance of FNN over NN. FNN also attains better performance than KNN and GCTA due to its ability of modeling the complex effects of indivdual SNPs and their interactions.

**Table 2:** The result from the analysis of two phenotypes, the pack-years of smoking and diabetes, using FNN, NN, KNN, and GCTA

	**Testing dataset**		**Training dataset**	
	**MSE/MCE**	**AUC/Cor**	**MSE/MCE**	**AUC/Cor**
**The pack-years of smoking**				
**FNN**	0.8753	0.3487	0.8590	0.3778
**NN**	0.9192	0.2806	0.8314	0.4686
**KNN**	0.9929	0.0579	0.9513	0.1096
**GCTA**	0.9926	0.0561	0.9557	0.0868
**Diabetes**				
**FNN**	0.0520	0.6080	0.0525	0.6482
**NN**	0.0520	0.5219	0.0525	0.5453
**KNN**	0.0489	0.5603	0.0490	0.6699
**GCTA**	0.0516	0.5555	0.0522	0.5878

We further evaluated the models for diabetes based on the misclassification rate and AUC. The FNN model obtained AUC values of 0.6482 and 0.6080 in training and testing data, outperforming the NN model, which obtained AUC values of 0.5453 and 0.5219 in training and testing data. The misclassification rates for both FNN and NN in the training and testing data were 0.0525 and 0.0520, respectively, closely aligning with the diabetes prevalence rate of 0.0524.

## Conclusion

We developed a C++ package, AIGen, a comprehensive AI software package for complex genetic data analysis. Based on two newly developed neural networks (i.e. kernel neural networks and functional neural networks), AIGen exhibits powerful and robust performance when dealing with complicated genotype-phenotype relationships (e.g. interactions) and high-dimensional genetic data. The integration with efficient algorithms, such as minimum norm quadratic unbiased estimation methods (MINQUE) and batch training, further enhances AIGen’s computational efficiency, which enables it to manage and analyze large-scale genetic data analysis with fast speed and high accuracy. As demonstrated by real data applications, AIGen has the ability to efficiently analyze biobank-scale genetic datasets with nearly half a million samples and SNPs. We have also shown that FNN and KNN attained higher accuracy than commonly used NN. While the performances of the formed genetic risk prediction models are limited by the heritability of the phenotypes (e.g. genetic variants only contribute to approximately $50\%$ of nicotine dependence variation), the models can be further improved once additional data (e.g. omics) become available in UKB. The issue of imbalanced data can occur for some phenotypes (e.g. diabetes). Under-sampling and resampling approaches can be used to reduce the imbalanced condition[19].

AIGen is built on two new neural network frameworks. While both KNN and FNN can be used for genetic data analysis, they are developed for different analytical needs. KNN is developed for the analysis involving a large number of genetic variants (e.g. genome-wide risk prediction analysis) and scalar phenotypes. Similarly to NN, FNN is not designed for genome-wide data analysis. Nevertheless, FNN can account for LD and be applied to various phenotypes, including high-dimensional phenotypes [12].

In conclusion, AIGen is an advanced AI tool for high-dimensional genetic data analysis, which can facilitate new findings and improve our understanding of complex genetic etiology. As we continue to refine and expand its features, we hope AIGen can play an important role in the new era of AI-based genetic research.

**Key Points**

- We developed a C++ package, AIGen, a computationally efficient and powerful AI software package for complex high-dimensional genetic data analysis;
- The C++ package integrates two newly developed neural networks, kernel neural networks and functional neural networks;
- We demonstrate the computational efficiency and accuracy performance of AIGen via the analysis of the UK Biobank dataset.

## Supplementary Material

AIGen__Supplementaryfile_bbae566
