# Supplementary material for: AIGen: an artificial intelligence software for complex genetic data analysis
Source: Brief Bioinform. 2024 Nov 16;25(6):bbae566. doi: 10.1093/bib/bbae566 (PMC11568876; doi:10.1093/bib/bbae566)
Supplement: AIGen__Supplementaryfile_bbae566 [file aigen__supplementaryfile_bbae566.pdf]

AIGen: An Artificial Intelligence Software for Complex Genetic Data Analysis

Tingting Hou[1], Xiaoxi Shen[2], Shan Zhang[3], Muxuan Liang[3], Li Chen[3], and Qing Lu[3, *]

[1]Department of Experimental Statistics, Louisiana State University, 45 Martin D. Woodin Hall, Baton Rouge, 70802, Louisiana, USA, [2]Department of Mathematics, Texas State University, 601 University Drive, San Marcos, 78666, TX, USA, and [3]Department of Biostatistics, University of Florida, 2004 Mowry Road, Gainesville, 32611, Florida, USA

[*]Corresponding author. lucienq@ufl.edu

Supplementary Data

1 Data distribution

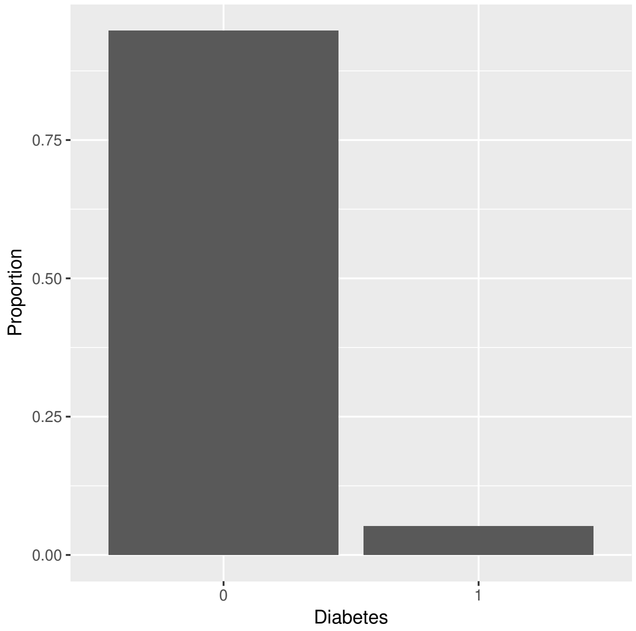

Figure 1: Percentage of Diabetes patients in the UK Biobank (UKB) Cohort. The bar plot illustrates the distribution of diabetes status among participants in the UK Biobank. The x-axis indicates the diabetes status, with ”0” representing participants without diabetes and ”1” representing those with diabetes. The y-axis shows the proportion of individuals within each group. The plot reveals a substantial imbalance, with a significantly larger proportion of participants without diabetes compared to those with diabetes.

2 Model Selection

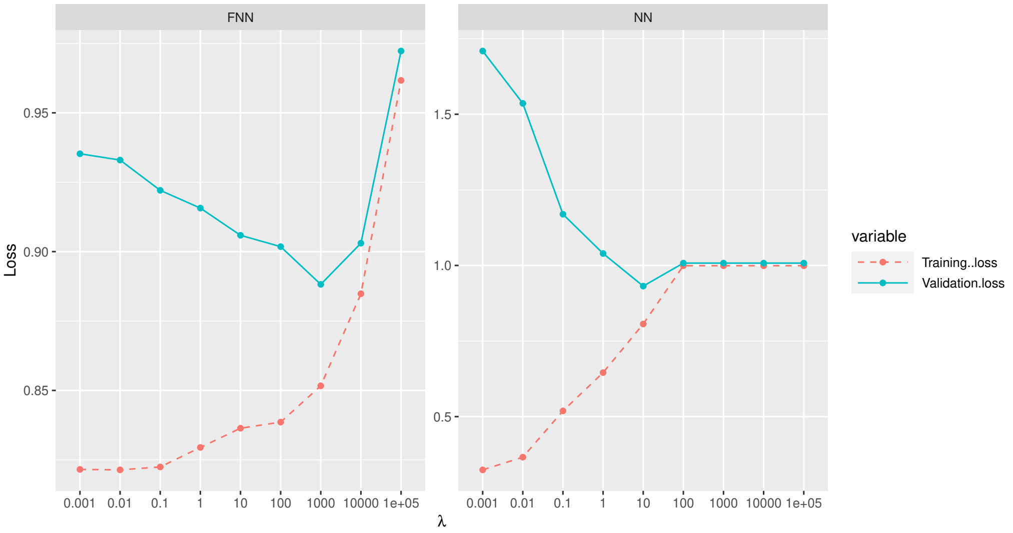

Figure 2: The selection of λ for the pack-years of smoking using functional neural network (FNN) and neural network (NN). On the left is a plot for FNN, and on the right is a plot for NN. Both plots graph the training loss (red line) and validation loss (blue line) across different values of λ whose values range from 0.001 to 10^5. The optimal λ for FNN is 1000 where the validation loss reaches its minimum whereas the optimal λ for NN is 10.

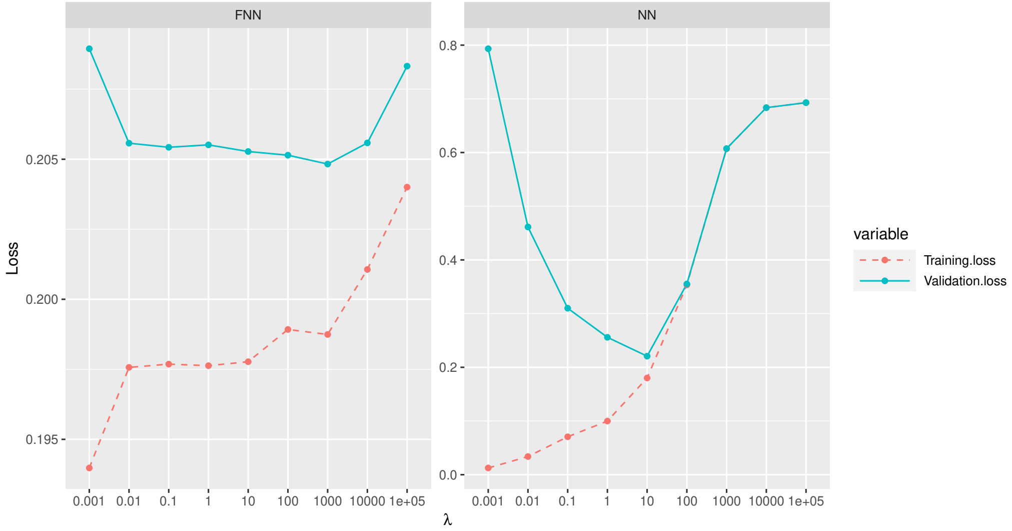

Figure 3: The selection of λ for diabetes using functional neural network (FNN) and neural network (NN). On the left is a plot for FNN, and on the right is a plot for NN. Both plots graph the training loss (red line) and validation loss (blue line) across different values of λ whose values range from 0.001 to 10^5. The optimal λ for FNN is 1000 where the validation loss reaches its minimum whereas the optimal λ for NN is 10.

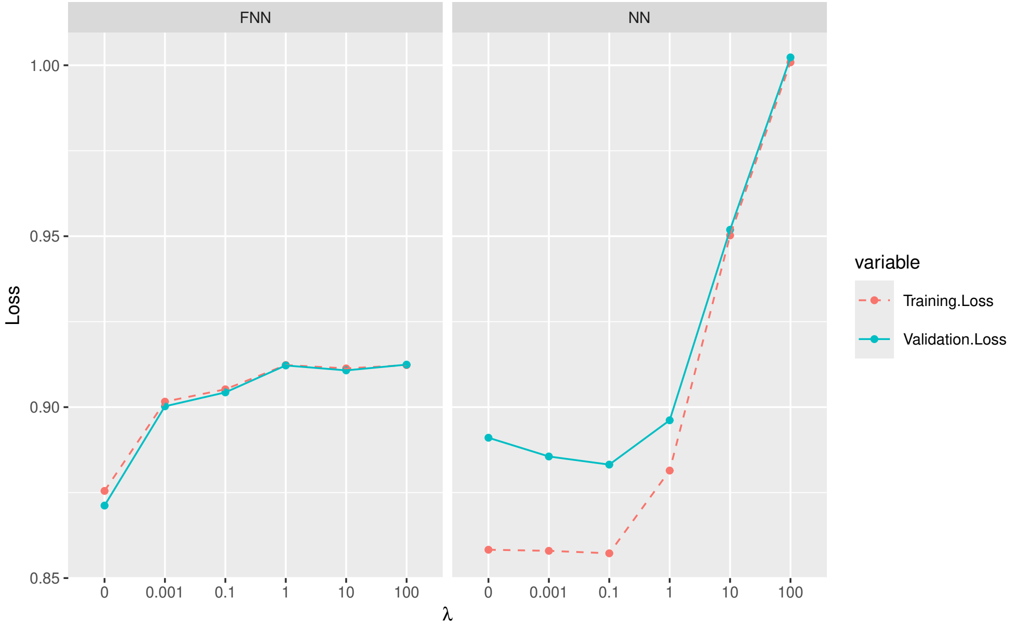

Figure 4: The selection of λ for the systolic blood pressure using functional neural network (FNN) and neural network (NN). On the left is a plot for FNN, and on the right is a plot for NN. Both plots graph the training loss (red line) and validation loss (blue line) across different values of λ whose values range from 0 to 100. The optimal λ for FNN is 0 where the validation loss reaches its minimum whereas the optimal λ for NN is 0.1.

3 Data analysis for Systolic blood pressure

In addition to Diabetes and smoking, We also used FNN to study the role of a candidate gene *C21orf91* in systolic blood pressure, considering covariates such as age, gender, and the top five principal components (PCs) [1]. Using Ensembl and PLINK, we extracted 13 SNPs in *C21orf91* from the UK Biobank genetic data and imputed missing values. The resulting dataset was split into training (80%) and testing (20%) sets. The training set was further divided into sub-training (80%) and validation (20%) sets.

We utilized 4th-order B-spline basis functions, with two hidden layers comprising 50 and 20 knots, and trained the functional neural network (FNN) over 100,000 epochs. The optimization was carried out using the 'adam' algorithm, and the optimal penalty parameter, λ, was selected based on the validation loss. Model performance was assessed on the testing data using mean squared error (MSE) and correlation metrics. For comparison, a traditional neural network (NN) with a similar structure, KNN and GCTA were also applied to the same data.

The MSE and correlation values for all methods are summarized in Table 1. Results from the testing data indicate that FNN outperformed the other three methods with a lower MSE and a higher correlation. Additionally, the MSE and correlation values from FNN were more consistent between the training and testing datasets compared to NN, suggesting a more robust performance of the FNN. The FNN also achieved better results than KNN and GCTA due to its capacity to model the complex effects of individual SNPs and their interactions.

Table 1: The result from the analysis of systolic blood pressure using FNN, NN, KNN and GCTA

	**Testing dataset**		**Training dataset**	
	**MSE/MCE**	**AUC/Cor**	**MSE/MCE**	**AUC/Cor**
**Systolic blood pressure**				
**FNN**	0.8707	0.3539	0.8743	0.3560
**NN**	0.8821	0.3379	0.8624	0.3727
**KNN**	0.8868	0.0040	0.8712	0.0827
**GCTA**	0.8866	0.0089	0.8758	0.0333

References

[1] Ervin R Fox, J Hunter Young, Yali Li, Albert W Dreisbach, Brendan J Keating, Solomon K Musani, Kiang Liu, Alanna C Morrison, Santhi Ganesh, Abdullah Kutlar, et al. Association of genetic variation with systolic and diastolic blood pressure among african americans: the candidate gene association resource study. *Human molecular genetics*, 20(11):2273–2284, 2011.
